# On Asthenopia, or Weaksightedness

**Published:** 1845-10

**Authors:** 


Art. I.—1. *On Asthenopia, or Weaksightedness.* By Wm. Mackenzie, m.d. Surgeon Oculist in Scotland in Ordinary to her Majesty, &c. (*From the Edinburgh Medical and Surgical Journal,* No. 156.)—*Edinb.* 1843. 8vo, pp. 34.

2. *On the Vision of Objects on and in the Eye.* By Wm. Mackenzie, m.d. &c. (*From the Edinburgh Medical and Surgical Journal,* No. 164.) —*Edinburgh,* 1845. 8vo, pp. 62.

By investigating the subjects of weaksightedness and muscæ volitantes in the elaborately systematic and scientific manner he has done in these papers, Dr. Mackenzie has imparted clearness and precision to what was before sufficiently puzzling and unsatisfactory, and has thus conferred a service on ophthalmic medicine. We shall here content ourselves by giving a brief outline of the facts and doctrines established by the author, referring the reader for further information to the original papers.

Asthenopia. The specific character of the weaksightedness which Dr. Mackenzie here considers, and to which he gives the name of Asthenopia, (from α privative, σθένος strength, and ὤψ the eye,) is this: The eyes are unable to sustain any continued exercise upon near objects; although the patient on first viewing them generally sees them distinctly, although he can employ his sight for any length of time in viewing distant objects, and although the eyes appear sound.

There is an incapacity to continue to converge the axis of the eyes on near objects, and for the pupil to continue contracted.

The diseases with which *asthenopia* is most apt to be confounded are photophobia, incipient myopia, presbyopia, night-blindness, and amblyopia or incomplete amaurosis. Asthenopia may also be complicated with other affections of vision—among others with muscæ volitantes.

The patients affected with asthenopia are generally delicate, but the complaint does not appear to be particularly connected with any disorder of the digestive organs, nor in females with disturbed menstruation. It commences in childhood or youth, and may continue throughout life. It is seldom met with originating in the middle period of life.

Asthenopia Dr. Mackenzie considers to be an affection of the apparatus by which the eye is adjusted for vision of near objects with some degree of affection of the retina.

The remote causes of asthenopia are very various: over-exertion of the sight, previous inflammation, especially scrofulous, of the eye, injuries of the fifth pair, affections of the encephalon, loss of the fluids of the body, &c. are enumerated. Though asthenopia does not run into amaurosis, it in general remains habitual, little or not at all influenced radically by treatment. It may, however, be palliated by resting the eyes at intervals, by the use of convex glasses; and when the patient's employment is one requiring much use of the eyes, he must change it for one of an opposite kind. "Many a poor man," says Dr. Mackenzie, "have I told to give up his sedentary trade and drive a horse and cart; while to those in better circumstances and not far advanced in life, I have recommended emigration, telling them that though they never could employ their eyes advantageously in reading and writing, they might see sufficiently to follow the pastoral pursuits of an Australian colonist."

MUSCÆ VOLITANTES. The appearances seen before the eyes, known under the name of *muscæ,* are of two principal kinds, such as have both apparent and real motion, and such as have apparent motion only—motion depending on that of the eye itself. These two kinds of muscæ are distinguished by the names of *floating* and *fixed,* and are quite different in their nature.

*Floating muscæ.* These are the most common kinds of muscæ. Overlooking the real motion which these muscæ present, some have viewed them as subjective sensations, depending on some intrinsic change of state of the optic nervous apparatus. That they are truly objective sensations, however,—occasioned by the presence of particles in the interior of the eye indeed, but extrinsic of and in front of the retina,—admits of mathematical demonstration. But more than this: the particles appear to be of normal occurrence in the eye, for the appearance of floating muscæ may in general be seen by any person, by simply looking through a small aperture in a card at the clear sky, or through the eyeglass of a compound microscope at the flame of a candle two or three feet distant.

On contemplating the spectra thus brought into view, viz. the beaded filaments, the distinctly and indistinctly-defined globules, and the watery-like filaments—called by Dr. Mackenzie, respectively, the *pearly spectrum,* the *distinct insulo-globular spectrum,* the *indistinct insulo-globular spectrum,* and the *watery spectrum*—it is observed that they are situated in different planes one behind the other, "that they never mingle with one another so as to change the order in which they stand before the eye, but the pearly spectrum always appears the nearest, then the sharply-defined insulo-globular, then the obscurely-defined globules, and farthest away the watery threads.

*Seat of the particles, the presence of which occasions floating muscæ.* A spectrum, like opake spots, surrounded by a halo, which occasionally seem to run together into dots, which again divide and disappear, and which ascend after every nictitation, which is sometimes seen and which appears to be produced by the layer of mucus and tears on the cornea—called therefore by Dr. Mackenzie, *muco-lachrymal muscæ*—has been confounded with floating muscæ, and the latter attributed to the same cause. That the particles which occasion floating muscæ, however, are situated in or behind the vitreous body, but in front of the retina, admits of being mathematically demonstrated, as also that they occupy different situations—those producing the pearly spectrum being the nearest to the retina, those producing the watery spectrum the farthest from the retina, the insulo-globular, intermediate.

As to the nature of the particles: this admits of less satisfactory determination than their existence and seat.

The action on the light by the particles, whatever they may be, which cause muscæ, appears to be diffraction or inflexion.

Though floating muscæ thus depend on a cause extrinsic to the retina, their being ordinarily seen is owing to a morbid and excitable state of the retina,—a state, however, which has no necessary tendency to run into amaurosis.

*Fixed muscæ.* These appearances, which are in their nature amaurotic symptoms, never change their position either in regard to each other or to the optic axis. They have thus no real motion, but merely apparent motion depending on the motions of the eyeball. It often, however, requires some attention and power of observation on the part of the patient, to distinguish real from apparent motion.

Fixed muscæ vary in number, size, and form. At first semi-transparent, they afterwards become black or at least dark. They appear like blotches when the patient looks at a sheet of white paper. Fixed muscæ are owing to spots of the retina becoming insensible. The insensible spots are apt to increase in size gradually until the whole retina is overspread with insensibility—is amaurotic.

Examples of temporary fixed spectra depending on natural states of the eye are the vascular spectrum in Purkinje's experiment, and the phenomena of accidental colours and ocular spectra.

